# Splenic Diffuse Red Pulp Small B-cell Lymphoma: A Rare Diagnosis in a Patient With Incidental Abnormal Lymphocytosis

**DOI:** 10.7759/cureus.85015

**Published:** 2025-05-29

**Authors:** Aarish Lalani, Roshniben Patel, Osama Mohiuddin, Abhishek Kalla, Laxman Wagle

**Affiliations:** 1 Internal Medicine, Ascension Saint Agnes Hospital, Baltimore, USA; 2 Hematology and Oncology, Ascension Saint Agnes Hospital, Baltimore, USA

**Keywords:** bone marrow, hairy cell leukemia, hairy cell leukemia variant, non-hodgkin lymphoma, peripheral blood, peripheral smear, splenic diffuse red pulp small b-cell lymphoma, splenic marginal zone lymphoma

## Abstract

Splenic diffuse red pulp small B-cell lymphoma (SDRPL) is an extremely rare form of non-Hodgkin lymphoma that has an indolent course and is characterized by splenomegaly, lymphocytosis, and hemocytopenia. Lymphomas with villous morphology are rare, with the least common subtype being SDRPL. Therefore, the diagnosis of SDPRL primarily hinges on a rigorous exclusion of other lymphoproliferative disorders, alongside a detailed correlation of histopathological features from bone marrow and spleen tissues, complemented by comprehensive immunophenotypic analysis. Adequate diagnosis is important to guide patient treatment and outcome. Here, we present a case of a 66-year-old male who presented with leukocytosis. At first, the patient was asymptomatic, though a peripheral blood smear revealed abnormal lymphocytes, and the presence of hairy cells led to a misdiagnosis of hairy cell leukemia (HCL), hence, no treatment was administered for six years. Subsequently, the patient was re-diagnosed with a splenic condition resembling a hairy cell variant, which later was found to be SDPRL. After minimal to no response on rituximab monotherapy, the patient was transitioned to cyclophosphamide and eventually to zanubrutinib, with subsequent hematologic improvement. This case underscores the diagnostic complexity of SDRPL, the importance of a multidisciplinary approach in distinguishing it from other splenic lymphomas, and highlights the therapeutic challenges in managing this rare entity.

## Introduction

Splenic diffuse red pulp small B-cell lymphoma (SDRPL) is a sporadic form of non-Hodgkin lymphoma (NHL). It was initially classified under the "Splenic B-cell Lymphoma/Leukemia Unclassifiable" group in the 2008 and 2016 World Health Organization (WHO) classifications of lymphoid neoplasms. However, it is now being classified under the "Mature B-cell neoplasms - Splenic B-cell lymphomas and leukemias" in the 2022 fifth edition of the WHO Classification of Haematolymphoid Tumours with an addition of a new entity, "splenic B-cell lymphoma/leukemia with prominent nucleoli (SBLPN)," which replaces the previous term “hairy cell leukemia (HCL) variant", in recognition that this proliferation is biologically distinct from HCL, although the leukaemic cells may partly resemble the “hairy cells” of HCL [1,2]. SDPRL is characterized by bone marrow involvement, splenomegaly, and circulating hairy cells manifesting as a leukemic process. Notably, substantial morphological overlap exists between SDRPL and other splenic lymphomas such as splenic marginal zone lymphoma (SMZL), HCL, and the less common hairy cell leukemia variant (HCL-V), which all present with villous lymphocytes [3]. The significant phenotypic similarities between these entities complicate accurate diagnosis. Although SDRPL is generally considered to be an indolent malignancy with transformation to aggressive lymphoma rarely being reported, precise distinction from other lymphoproliferative disorders is essential for guiding therapeutic management.

## Case presentation

A 66-year-old White male with a history of hypertension and hyperlipidemia initially presented to the hematology clinic after leukocytosis was noted on a peripheral blood smear (PBS) during a recent hospitalization. The patient had sustained a mechanical fall resulting in a forehead laceration and was evaluated in the emergency department (ED). Laboratory studies, including a complete blood count (CBC) with smear, were performed, and he was discharged home. Subsequent review of the PBS revealed abnormal lymphocytes concerning for leukemia or lymphoma. The patient was advised to follow up in the outpatient clinic for additional workup, including flow cytometry. At this visit, he reported experiencing frequent night sweats with waxing and waning intensity over several months. He denied fever, chills, lightheadedness, fatigue, weakness, or changes in appetite or weight.

**Figure 1:**
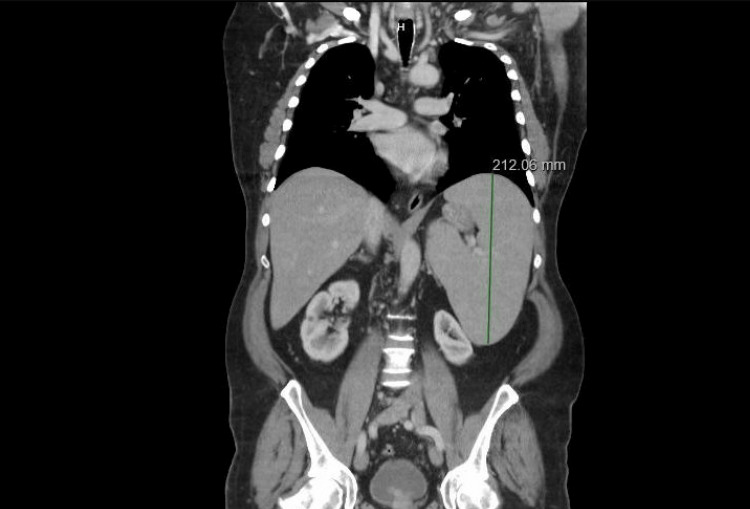
CT scan of the chest, abdomen, and pelvis showing hepatomegaly and splenomegaly, with the spleen measuring 21.2 cm.

Flow cytometry results were consistent with HCL based on the presence of abnormal B cells and immunophenotype typical of HCL (bright intensity CD20 and CD11c with coexpression of CD103, although CD25 was negative, which is often positive in hairy cell). His white blood cell (WBC) count was 19.3 × 10^3^/µL (normal range: 4-11 × 10^3^/µL), hemoglobin (Hb) was 16 g/dL (normal range: 12-15 g/dL), and platelet count was 189 × 10^3^/µL (normal range: 125-490 × 10^3^/µL) (see Table 1 for details). A follow-up computed tomography (CT) scan of the chest, abdomen, and pelvis was performed for staging. It revealed splenomegaly (21.2 cm), subcentimeter retroperitoneal and periaortic lymphadenopathy, and a right common iliac chain lymph node measuring 11 × 9 mm (Figure 1).

**Table 1:** Complete blood count (CBC) results from initial presentation, and before and after initiation of cycle 1 of rituximab therapy.

CBC	First Visit	Before Cycle 1 of Rituximab Therapy			After Cycle 1 of Rituximab Therapy		
White blood cell count (4.0-11.0 K/uL)	19.3	37.1	45.2	51.2	86.6	45.6	74.3
Hemoglobin (13.0-17.0 g/dL)	16.0	12.5	13.5	12.3	12.3	11.8	10.4
Platelet count (150-400 K/uL)	189	126	141	115	149	48	71

The case was presented at a multidisciplinary tumor board. Since the patient was asymptomatic except for intermittent night sweats and stable leukocytosis, observation without treatment was recommended.

**Figure 2:**
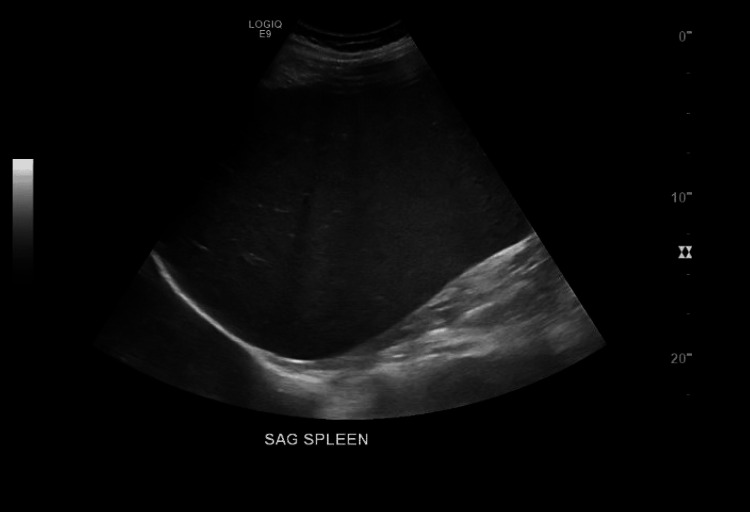
Abdominal ultrasound showing splenomegaly of 25 cm. SAG: sagittal

The patient was monitored over the next six years, with follow-up visits every three months initially, then every six months. Serial laboratory and imaging studies remained stable, and no treatment was initiated during this period. In January 2024, repeat blood work revealed an increase in WBC count to 37.1 × 10^3^/µL (normal range: 4-11 × 10^3^/µL), along with mild anemia and thrombocytopenia. An abdominal ultrasound demonstrated hepatosplenomegaly, with the liver measuring 23.6 cm (with normal parenchymal echogenicity) and the spleen measuring 25 cm, an increase from prior measurements (Figure 2).

**Figure 3:**
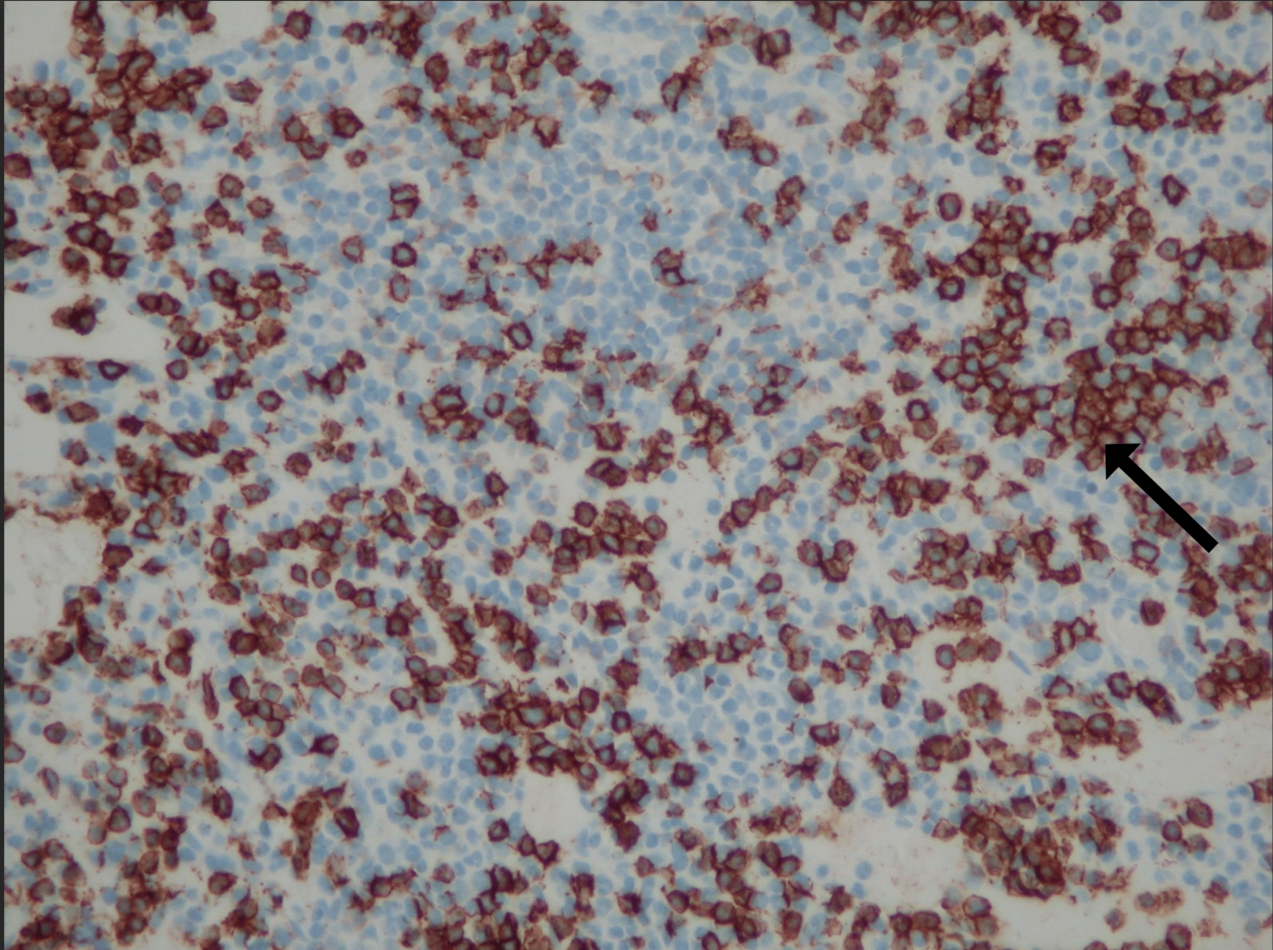
Immunohistochemistry staining demonstrating aggregates of CD3+ T cells and a diffuse interstitial population of CD20+ B cells (black arrow).

**Figure 4:**
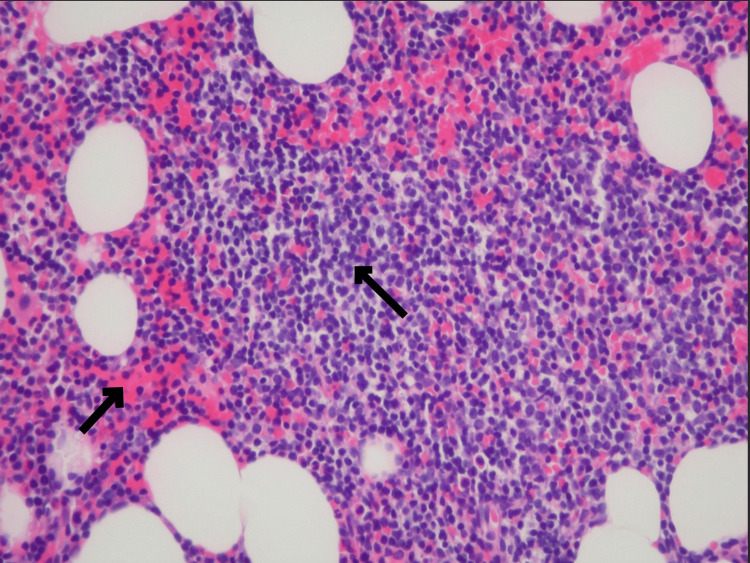
Hematoxylin and eosin (H&E) stain demonstrating increased marrow cellularity with several lymphoid aggregates. Reticulin stain showing a patchy increase in reticulin fibers (black arrows).

A bone marrow biopsy revealed a CD103-positive, CD25-negative, lambda light chain-restricted mature B-cell lymphoproliferative neoplasm with focal reticulin fibrosis. BRAF mutation testing was negative, and cytogenetic analysis showed an abnormal karyotype with a t(1;5) translocation in five cells (Figures 3-4).

After further discussion with a pathologist and review at a hematology case conference, the diagnosis of an HCL-v was initially considered due to the presence of CD103-positive but CD25-negative cells. However, based on the biopsy results and overall findings, the best classification was deemed a mature B-cell lymphoproliferative neoplasm. A positron emission tomography (PET) scan was performed to assess for Richter transformation. No fluorodeoxyglucose (FDG) uptake was seen in the spleen or lymph nodes, although the scan revealed splenomegaly (28 cm), hepatomegaly (23 cm), and numerous small, non-FDG-avid pulmonary nodules bilaterally. The reduced FDG avidity was attributed to recent corticosteroid use for symptomatic relief.

The patient was started on rituximab at 25 mg/m^2^ intravenously (IV) once weekly, along with allopurinol for tumor lysis syndrome prophylaxis. Following the first dose of the second cycle of rituximab treatment, the patient was admitted to the hospital with chills and was found to have a severely elevated WBC count up to 133.4 x 10^3^/µL (normal range: 4-11 × 10^3^/µL) (Table 2). Infectious causes were all ruled out, and the patient remained asymptomatic otherwise. It was concluded that the WBC spike likely reflected a therapy-induced lymphocyte shift from the spleen to the peripheral circulation.

**Table 2:** Complete blood count (CBC) results before and after initiation of cycle 2 rituximab therapy.

CBC	Before Cycle 2 of Rituximab Therapy			After Cycle 2 of Rituximab Therapy		
White blood cell count (4.0-11.0 K/uL)	27.0	36.1	31.0	133.4	96.8	40.3
Hemoglobin (13.0-17.0 g/dL)	11.4	10.9	10.7	10.6	9.5	10
Platelet count (150-400 K/uL)	139	141	41	86	83	89

**Figure 5:**
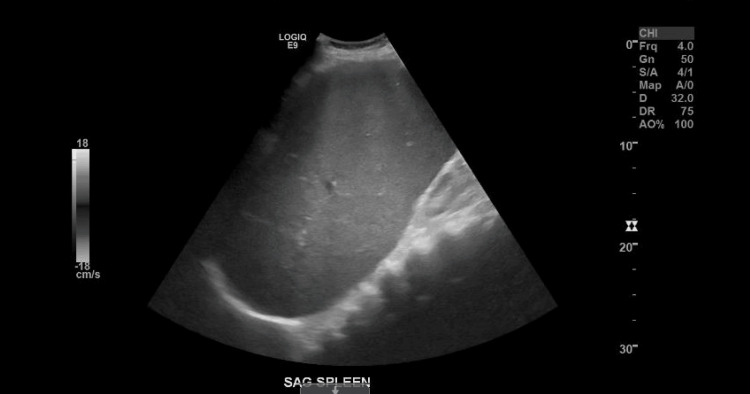
Abdominal ultrasound showing splenomegaly of 29 cm. SAG: sagittal

However, follow-up abdominal ultrasound showed persistent hepatomegaly (23.3 cm) and increased splenomegaly (29 cm; previously 25 cm), despite rituximab therapy (Figure 5).

Following an extensive literature review and multidisciplinary consultation, the clinical and pathological features, including bone marrow biopsy findings of CD103+/CD25- but absence of BRAF and lack of BRAF V600E mutation, were deemed most consistent with SDRPL. The patient was advised to undergo four additional rituximab infusions.

**Figure 6:**
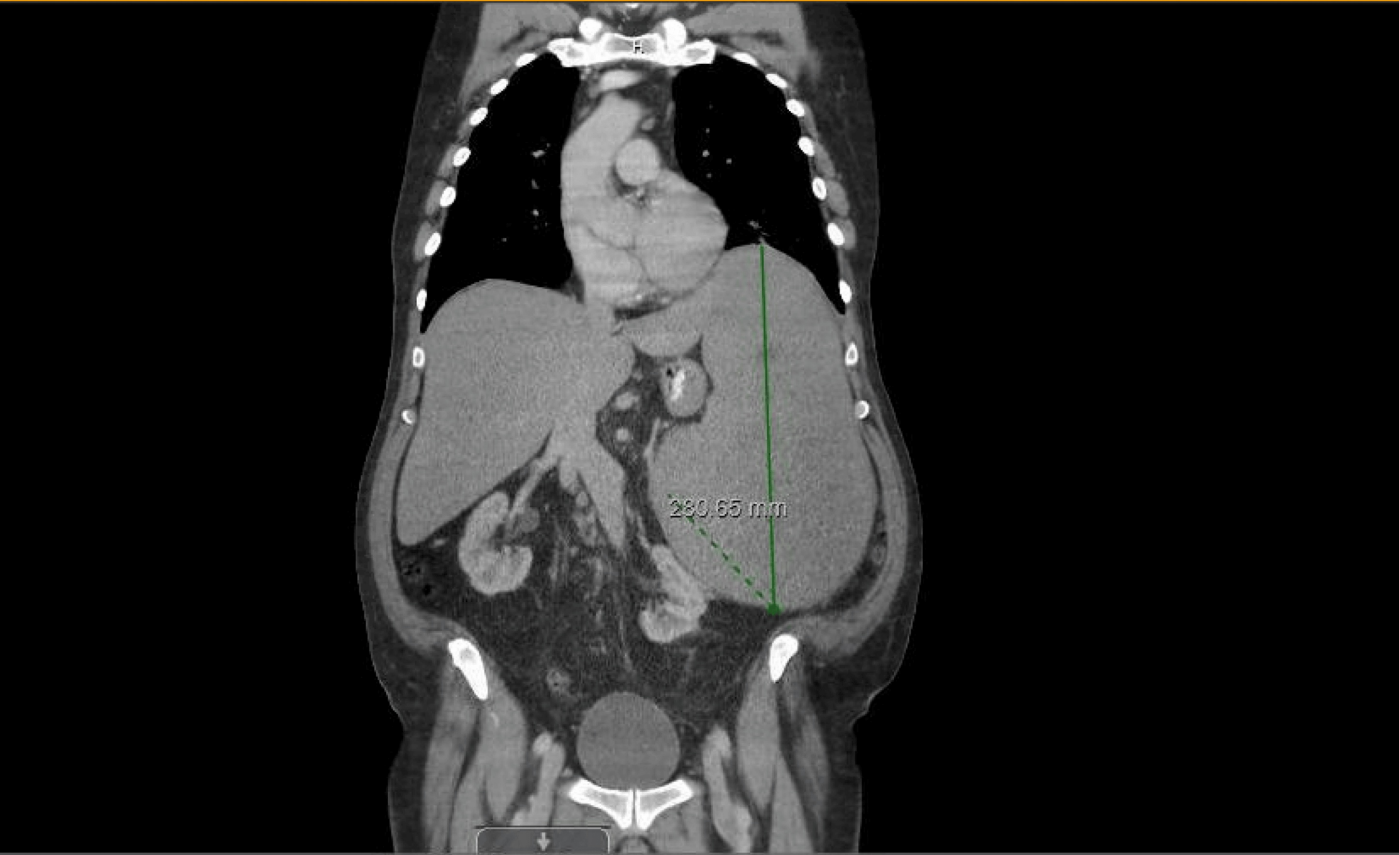
CT scan of the chest, abdomen, and pelvis showing hepatomegaly and splenomegaly, with the spleen measuring 28 cm.

After completing two cycles of rituximab, a repeat CT of the abdomen and pelvis (Figure 6) showed mild hepatomegaly (22 cm) and worsening splenomegaly, now measuring 31 × 18 × 28 cm (volume 8124 cc), compared to a prior size of 27 × 16 × 28 cm (volume 6289 cc).

Due to minimal hepatic improvement and worsening splenomegaly, the patient was started on cyclophosphamide at 750 mg/m^2^. Following the first dose of cyclophosphamide treatment, the patient was admitted to the hospital again with fever and fatigue and was again found to have a severely elevated WBC count up to 106.3 K/uL (normal range: 4-11 K/uL) (Table 3). Infectious causes were negative, the patient remained asymptomatic otherwise, and his symptoms were considered to be a result of hypersensitivity pneumonitis.

**Table 3:** Complete blood count (CBC) results before and after initiation of cyclophosphamide therapy.

CBC	Before Cyclophosphamide Therapy			After Cyclophosphamide Therapy		
White blood cell count (4.0-11.0 K/uL)	51.1	48.7	91.2	106.3	66.7	52.5
Hemoglobin (13.0-17.0 g/dL)	9.6	9.5	9.0	8.8	8.4	7.7
Platelet count (150-400 K/uL)	125	112	108	91	70	62

At post-discharge follow-up, the patient’s hematologic parameters had improved: WBC 43.2 × 10^3^/µL, Hb 10.5 g/dL (up from a nadir of 6.7 g/dL), mean corpuscular volume (MCV) 87.7 fL (nadir 80.8 fL), and platelet count 69 × 10^3^/µL (previously 35 × 10^3^/µL). Due to continued clinical improvement and residual thrombocytopenia, cyclophosphamide was discontinued, and zanubrutinib was initiated at 160 mg twice daily after another hematology conference meeting, due to overlapping features with marginal zone lymphoma, SDRPL, and HCL.

At the three-month follow-up, the patient remained on zanubrutinib with continued hematologic improvement: WBC 12 × 10^3^/µL, Hb 13.4 g/dL, MCV 86.4 fL, and platelet count 122 × 10^3^/µL (Table 4). Repeat imaging was planned to assess for further resolution of hepatosplenomegaly. 

**Table 4:** Complete blood count (CBC) at two months post-discharge showing improvement in cell counts.

CBC	Post-discharge Outpatient Visit		
White blood cell count (4.0-11.0 K/uL)	16.5	9.6	12
Hemoglobin (13.0-17.0 g/dL)	11.7	12.3	13.4
Platelet count (150-400 K/uL)	104	81	122

## Discussion

SDRPL is a rare subtype of B-cell lymphoma characterized by diffuse infiltration of the bone marrow, peripheral blood, and splenic red pulp by small, monomorphic B lymphocytes [4]. SDRPL is typically a diagnosis of exclusion. The differential diagnosis includes SMZL, HCL, and HCL-v [4]. The true incidence of SDRPL is unknown due to limited available studies [4]. SDRPL accounts for less than 1% of NHLs and less than 10% of B-cell lymphomas diagnosed via splenectomy [4,5]. It shows a slight male predominance, with a male-to-female ratio of approximately 1.5-2.5:1 [4,6]. Most patients are over 60 years old, with a median age of 65 years, in contrast to the more even gender distribution or slight female predominance observed in SMZL [4,6].

Clinical manifestations of SDRPL include massive splenomegaly, often causing abdominal pain [4,6]. B symptoms, such as fever, night sweats, and unintentional weight loss greater than 10%, occur in up to one-third of patients [4,6]. Leukopenia and thrombocytopenia are usually mild, while anemia is rare [4]. Two case reports have documented concurrent chronic hepatitis B infection in patients with SDRPL [4]. PBSs typically show a homogeneous infiltrate of small to medium-sized cells with eccentrically placed nuclei, basophilic cytoplasm, and characteristic cytoplasmic projections [6-9]. Hilar splenic lymph nodes are commonly involved, whereas peripheral lymph nodes are rarely affected [8,9]. CT imaging reveals diffuse splenic enlargement without discrete lesions [4], while positron emission tomography-computed tomography (PET-CT) may demonstrate mildly increased, homogeneous FDG avidity in the spleen [4]. Occasionally, PET-CT shows normal spleen size and uptake. Rarely, transformation to diffuse large B-cell lymphoma, marked by elevated lactate dehydrogenase (LDH) levels, a high Ki-67 index, and aggressive clinical behavior, or transformation to B-cell prolymphocytic leukemia, has been reported [4].

Definitive diagnosis requires splenic biopsy or splenectomy, which reveals diffuse infiltration of both the splenic red and white pulp [6,8,9]. Bone marrow involvement shows variable cellularity, preservation of hematopoietic reserve, and mild fibrosis [8]. Immunophenotyping typically demonstrates expression of CD20 and DBA.44, with cells frequently IgG-positive. Negative markers include CD25, annexin A1, cyclin D1, CD103, CD123, and CD27 [8-10]. Cytogenetic abnormalities such as 7q deletion, trisomy 18, and 17p deletion may be observed [10].

Table 5 compares the key immunophenotypic markers (CD5, CD10, CD23, CD25, CD103, cyclin D1) to differentiate chronic lymphocytic leukemia (CLL), mantle cell lymphoma (MCL), follicular lymphoma (FL), HCL, and SDRPL.

**Table 5:** Comparison of key immunophenotypic markers (CD5, CD10, CD23, CD25, CD103, cyclin D1) to differentiate chronic lymphocytic leukemia (CLL), mantle cell lymphoma (MCL), follicular lymphoma (FL), hairy cell leukemia (HCL), and splenic diffuse red pulp lymphoma (SDRPL).

Disease	CD5	CD10	CD23	CD25	CD103	Cyclin D1	Other Key Markers
CLL	+	−	+	+	−	−	CD19+, CD20 dim, CD200+
MCL	+	−	−	−	−	+	CD19+, CD20+, SOX11+
FL	−	+	−	−	−	−	BCL2+, BCL6+, CD19+, CD20+
HCL	−	−	−	+	+	−	CD11c+, CD123+, Annexin A1+, TRAP+
SDRPL	−	−	−	−	±	−	CD11c+, DBA44+, cyclin D3 (variable)

Since this disease is uncommon, there are limited retrospective or prospective clinical studies that evaluate different treatment options [4]. Treatment strategies include watchful waiting, splenectomy, and rituximab monotherapy [4,8]. No studies to date have directly compared treatment modalities [4,8], and there is currently no standardized treatment approach [8,11]. Some reports suggest that combining splenectomy with chemotherapy may improve outcomes [11]. Due to the indolent nature of SDRPL, a “watch and wait” approach is preferred for asymptomatic patients [4]. For symptomatic individuals, splenectomy and rituximab monotherapy are the most commonly employed treatments [4]. Chemotherapy is typically reserved for progressive disease [4]. While splenectomy is not curative, since residual disease often remains in the bone marrow and peripheral blood, it can induce remission. However, it carries risks of postoperative complications and infection [4]. Rituximab monotherapy is generally well-tolerated and is commonly recommended, especially for older patients [4,12]. Several clinical trials have investigated the use of zanubrutinib in SDRPL, especially for patients who have not responded to other therapies, as in our case, or are at a high risk of disease progression.

SDRPL is considered incurable, but it is associated with significantly longer progression-free survival compared to SMZL [4,8]. The five-year overall survival rate is approximately 93% [4,6]. Hb level below 10 g/dL is associated with a poorer prognosis [6], likely due to marrow infiltration and increased disease burden.

Zanubrutinib is a highly selective Bruton tyrosine kinase (BTK) inhibitor and is often chosen over other BTK inhibitors due to its improved efficacy, safety, and tolerability in various B-cell malignancies. Since SDRPL is a rare condition, there is no disease-specific clinical trial comparing different BTK inhibitors, however, this has been explored in other lymphocytic malignancies. The ALPINE trial compares zanubrutinib or ibrutinib in relapsed or refractory CLL, which showed longer progression-free survival of patients who received zanubrutinib than those who received ibrutinib, and zanubrutinib was associated with fewer cardiac adverse events [13]. The ASPEN study, on the other hand, compared zanubrutinib versus ibrutinib in symptomatic Waldenström macroglobulinemia (WM), which showed that although zanubrutinib and ibrutinib are highly effective in the treatment of WM, zanubrutinib treatment was associated with a trend toward better response quality and less toxicity, particularly cardiovascular toxicity [14].

## Conclusions

SDRPL is a rare, indolent subtype of NHL that presents significant diagnostic and therapeutic challenges due to its clinical and morphological resemblance to other splenic lymphoproliferative disorders, particularly SMZL, HCL, and HCL-v. Our case, with its prolonged asymptomatic phase, initial misdiagnosis as HCL, and eventual progression, underscores the importance of careful histological review, immunophenotyping, and exclusion of similar lymphoproliferative disorders. This case also illustrates the unique challenge of managing SDRPL when standard treatment options fail. The patient’s eventual clinical improvement on zanubrutinib highlights the potential role of targeted therapies in refractory or progressive cases. As awareness of SDRPL increases, clinicians must remain vigilant in distinguishing it from other splenic lymphomas, especially when treatment decisions hinge on an accurate diagnosis. Ongoing research and reporting of such rare cases are essential to improving our understanding and guiding future management strategies.
